# The role of International Society of Paediatric Oncology (SIOP) in advancing global childhood cancer care

**DOI:** 10.3332/ecancer.2024.1678

**Published:** 2024-02-28

**Authors:** Julia Challinor, Alan Davidson, Guillermo Chantada, Rejin Kebudi, Kathy Pritchard-Jones

**Affiliations:** 1 School of Nursing, University of California San Francisco, 2 Koret Way, San Francisco, CA 94143, USA; 2 Pediatric Hematology-Oncology Service, Red Cross War Memorial Children’s Hospital, University of Cape Town, Rondebosch, Cape Town 7700, South Africa; 3 Department of Pediatric Oncology, Hospital Sant Joan de Deu, Pg de Sant Joan de Déu, 2, Esplugues de Llobregat, 08950 Barcelona, Spain; 4 Departamento de Montevideo, Fundación Perez Scremini, Bv Gral Artigas 1556, Montevideo 11600, Uruguay; 5 Division of Pediatric Hematology-Oncology, Department of Clinical Oncology, Oncology Institute, Topkapı, Turgut Özal Millet Cd No:118, 34093 Fatih/Istanbul, Turkey; 6 Division of Pediatric Hematology-Oncology, Department of Preventive Oncology, Oncology Institute, Topkapı, Turgut Özal Millet Cd No:118, 34093 Fatih/Istanbul, Turkey; 7 University College London Great Ormond Street Institute of Child Health, 30 Guilford St, WC1N 1EH London, UK; a https://orcid.org/0000-0002-5008-8501; b https://orcid.org/0000-0002-4646-4332; c https://orcid.org/0000-0002-9375-9336; d https://orcid.org/0000-0003-4344-8174; e https://orcid.org/0000-0002-2384-9475

**Keywords:** childhood cancer, low- and middle-income countries, education, advocacy, research

## Abstract

The Société Internationale d’Oncologie Pédiatrique [International Society of Paediatric Oncology] (SIOP), founded in 1969, aims to improve the lives of children and adolescents with cancer through global collaboration, education, training, research and advocacy. The annual congress provides the opportunity to share late-breaking research, clinical experiences and debate, with experts worldwide. SIOP’s six Continental Branches represent their constituent members in North America, Oceania, Latin America, Africa, Europe and Asia and bring best practices and recent research findings of value to their specific patient populations. In 1990, the SIOP Board of Directors addressed the formerly predominantly European/North American society transforming into a global association by establishing a scholarship program to bring low- and middle-income country (LMIC) paediatric oncologists and nurses to SIOP meetings. A major achievement was SIOP’s acceptance as a World Health Organisation (WHO) non-state actor in official relations in 2018, joining 220 non-governmental organisations, international business associations and philanthropic foundations with this privilege. SIOP supports advocacy with WHO member states and civil society to highlight the specific needs of cancer in this age-group through key programs especially supporting the WHO Global Initiative for Childhood Cancer. Sustained improvement in childhood cancer outcomes has paralleled the integration of research with care; thus, SIOP launched a Programme for Advancing Research Capacity for funding selected clinical trial groups in LMICs. SIOP supports south-south partnerships, and the principles elegantly expressed in SIOP Africa’s checklist for co-branding projects, that include the prioritisation of local needs, cultivation of local expertise and commitment to equitable partnerships. SIOP now counts approximately 3,000 members from over 128 countries; 39% are from more than 60 LMICs. SIOP members have multidisciplinary expertise on all aspects of childhood cancer care working in collaboration with key stakeholders including governments, civil society organisations and funders to improve the lives of children/adolescents with cancer everywhere in all ways.

The Société Internationale d’Oncologie Pédiatrique [International Society of Paediatric Oncology] (SIOP), founded by French physician, Odile Schweisguth, and colleagues in 1969, sought to promote the study and care of children with cancer. The first SIOP annual meeting in 1969 was held in Madrid, and subsequent meetings in various European cities. As intercontinental membership increased, cities from other continents hosted this meeting, which now rotates among continents. In the early years, two clinical trials (nephroblastoma and medulloblastoma) were planned during the SIOP Congress and were conducted by SIOP members. However, in the late 1980s, SIOP officially recognised that its role in clinical trials was best as a convener/facilitator not as an implementing organisation, thus leaving this latter responsibility to national or continental cooperative groups.

The SIOP annual Congress, held in collaboration with Childhood Cancer International (CCI) (parents and survivor groups), the Paediatric Radiation Oncology Society (PROS) and the International Society of Paediatric Surgical Oncology (IPSO) has been conducted in a hybrid format over the last two years to increase participation. This Congress provides the opportunity to share late-breaking research and clinical experiences and debate, discuss and learn from experts worldwide.

SIOP’s six Continental Branches represent their constituent members in North America, Oceania, Latin America, Africa, Europe and Asia. The last four conduct annual conferences (Africa is biannual) for their regions to bring best practices and recent research findings of value to their specific patient and family populations. Professor Hiroki Hori, SIOP Asia Continental President, notes that continental scientific meetings provide more opportunities for health personnel engaged in paediatric cancer practice to learn about advancements in the field and share the experiences with experts in regional countries in similar situations. Above all, people can easily access the scientific meetings in neighbouring countries, thus reducing the need for a visa to attend annual SIOP Congresses – an increasing challenge for low- and middle-income country (LMIC) attendees. Andrea Cappellano, SIOP Latin America Continental President (SLAOP – Sociedad Latinoamericana de Oncología Pediátrica [Latin American Society of Pediatric Oncology]) states that continental branches are essential for paediatric oncology collaboration, education and training, advocacy and awareness. SLAOP has designated Spanish and Portuguese as official languages, and thus enhanced its effectiveness and reach across Latin America, promoting better collaboration, information sharing and support among its diverse members and stakeholders.

## Global childhood cancer disparities

In a world facing climate change, conflict, unexpected disasters and uncertain economic and political circumstances, the disparity in access to childhood cancer care has become more visible. Despite efforts by the World Health Organisation (WHO) to promote universal health coverage and address the emerging burden of non-communicable diseases, and childhood cancer specifically through the Global Initiative for Childhood Cancer (GICC), a vast inequity in treatment options and survival remains. The insufficiency of an adequate specialised and acknowledged workforce, essential medicines and technologies and primary care professionals’ awareness of paediatric oncology limits the success of many facilities in LMICs where the vast majority of children and adolescents with cancer live. Facilities in high-income countries are experiencing shortages in critical chemotherapy, nursing staff and challenges in addressing social determinants of health to mitigate disparities in access to clinical trials, survivorship and follow-up care.

In 1990, the SIOP Board of Directors decided to transform the formerly predominantly European/North American Society into a global association. At that time, only 10% of the members were from LMICs so SIOP established a scholarship program to bring paediatric oncologists and nurses in LMICs to SIOP meetings. During the 2010 annual Congress in Boston, there was a ‘Restructuring Meeting’ of the Paediatric Oncology in Developing Countries (PODC) Committee, and 12 working groups were established. In 2021, the PODC was renamed as the Global Health Network and now focuses on relevant issues of childhood cancer such as inequities in access, resources, health systems and specialty care, e.g., radiotherapy and palliation.

## SIOP collaborations

A major achievement was SIOP’s acceptance as a non-state actor in official relations with the WHO in 2018. SIOP supports advocacy with WHO member states and civil society to highlight the specific needs of cancer in this age-group through key programs. Such advocacy covers both the cancer and paediatric agendas to ensure a more integrated approach to early diagnosis, essential medicines, therapeutic advances and holistic support throughout a child or adolescent’s life course*.* SIOP also collaborates with other UN agencies and the WHO GICC member states to offer coordinated support that national health systems and regional networks can draw upon to implement improvements in their childhood cancer services and patient outcomes.

SIOP has pioneered the global development of adapted treatment guidelines which later, in partnership with St Jude Children’s Research Hospital in the USA, IPSO, PROS and CCI became the Adapted Resource and Implementation Application guide that health care providers can use to create and monitor ‘standard of care’ protocols for all types of cancer that they treat according to their available resources. Working with the International Paediatric Association, SIOP created the Childhood Cancer Early Diagnosis and Appropriate Referral programme aimed at improving knowledge of primary and community healthcare workers, with more to follow. SIOP’s professional networks provide a range of independent expertise to support, for example, baseline standards for paediatric oncology nursing in LMICs [1] and pragmatic guidance on the expectations of LMICs in ‘twinning partnerships’ [2].

Sustained improvement in childhood cancer outcomes has paralleled the integration of research with care. To support this principle at a global level, SIOP launched its Programme for Advancing Research Capacity for paediatric cancer clinical trials in 2022 (https://siop-online.org/parc-committee/), with support from several philanthropic partners. The catalytic grants awarded to date have been focused on regional co-operative clinical research groups, recognising their importance as drivers of advancing knowledge adapted to local priorities. This aligns with strengthening relationships between the presidents and members of the SIOP’s Continental Branches and WHO regional cancer leaders, so regional resources and expertise can be applied most effectively, avoiding duplication and accelerating understanding of cancer outcomes in the local populations.

## SIOP’s global support and advocacy

The focus of twinning partnerships has historically been north-south, but there is an ever-increasing number of centres in the south where clinical platforms, educational systems and research capacity lend themselves to providing clinical support and training for regional partners. The principles underpinning SIOP Africa’s checklist for co-branding projects include the prioritisation of local needs, the cultivation of local expertise and a commitment to equitable partnerships. There can be no greater guarantee of same than the development of strong regional collaborations in the south. And the involvement of multidisciplinary team partners in PROS, IPSO and CCI from board level to the coalface strengthens networking and knowledge sharing and predicates for community involvement.

These principles are well illustrated by a number of regional training networks in Africa driven by SIOP and PROS members in southern (African Paediatric Fellowship Programme and Access to Care^1^) [3], eastern (Mulago National Referral Hospital in Uganda and Global Hope from Texas Children’s Hospital in the USA^2^), western (Ghana College and World Child Cancer,^3^ a foundation in the UK) and northern Africa (Groupe Franco-Africain d’Oncologie Pédiatrique [Francophone-African Group of Paediatric Oncology or GFAOP]*,*^4^ and Children’s Cancer Hospital in Egypt^5^). Contextually authentic training and professional development is possible and creates relationships and systems which lead to ongoing clinical support (with extensive use of telemedicine) and research collaborations. These programmes help to build local capacity and reduce dependency on foreign expertise. They are also a much better guarantee of skills retention.

From a research perspective south-south collaborations have led to the development of clinical trial groups such as Asociación Hemato-Oncológica Pediátrica de Centro América [Pediatric Hematology-Oncology Association of Central America],^6^ GFAOP, Grupo América Latina de Oncología Pediátrica [Latin American Pediatric Oncology Group],^7^ Indian Pediatric Hematology Oncology Group (IPHOG)^8^ and CANCaRe Africa,^9^ accruing children into clinical trials using adapted therapy regimes and reporting the results. There is also scope for co-operation between regional WHO structures and regional political groupings. A recent publication by SIOP Africa Continental members calls on the WHO African Region and the African Union to collaborate to advance the WHO GICC, and the cause of childhood cancer in general [4].

## Summary

For over 50 years, SIOP has served as an international forum for childhood cancer stakeholders including clinicians, scientists, researchers, parents, survivors and advocates, conducting work throughout the year, and convening at each multidisciplinary annual SIOP Congress to share best practices and progress. SIOP’s Continental Branch structure and congresses strengthen regional participation, contributions and collaboration. The SIOP Global Mapping Programme, now complete for two continents (Africa and Latin America) [5, 6] and moving forward on a third (Asia), will eventually enable us to locate and share a map with every centre and facility for paediatric cancer in the world. SIOP’s role in the WHO GICC is critical to galvanise local support for government action to address disparities in access to care, universal health coverage to include childhood cancer treatment as well as follow-up surveillance for survivors and to ensure essential medicines and technologies are available for all children and adolescents regardless of where they live. SIOP now counts approximately 3,000 members from over 128 countries, 39% of them from more than 60 LMIC. We are a Society with deep multidisciplinary expertise from around the world on all aspects of childhood cancer care working in collaboration with key stakeholders including governments, civil society organisations and funders to improve the lives of children/adolescents with cancer everywhere in all ways.

## Conflicts of interest

The authors declare no financial conflicts of interest.

## Funding

No funding was received for the preparation or submission of this manuscript.

## Footnotes

1
https://theapfp.org/

2
https://www.texaschildrensglobalhealth.org/global-hope

3
https://worldchildcancer.org/

4
https://www.gfaop.org/

5
https://www.57357.org/en/home-page/

6
https://www.facebook.com/ahopca/

7
https://grupogalop.org/

8
https://www.inphog.org/

9
https://siop-online.org/cancareafrica/
